# Supplementary material for: The causal relationship between the human gut microbiota and pyogenic arthritis: a Mendelian randomization study
Source: Front Cell Infect Microbiol. 2024 Nov 26;14:1452480. doi: 10.3389/fcimb.2024.1452480 (PMC11629706; doi:10.3389/fcimb.2024.1452480)
Supplement: Supplementary file 1 [file DataSheet1.zip › mendelian test/ebi-a-GCST90027743.csv_forest.pdf]

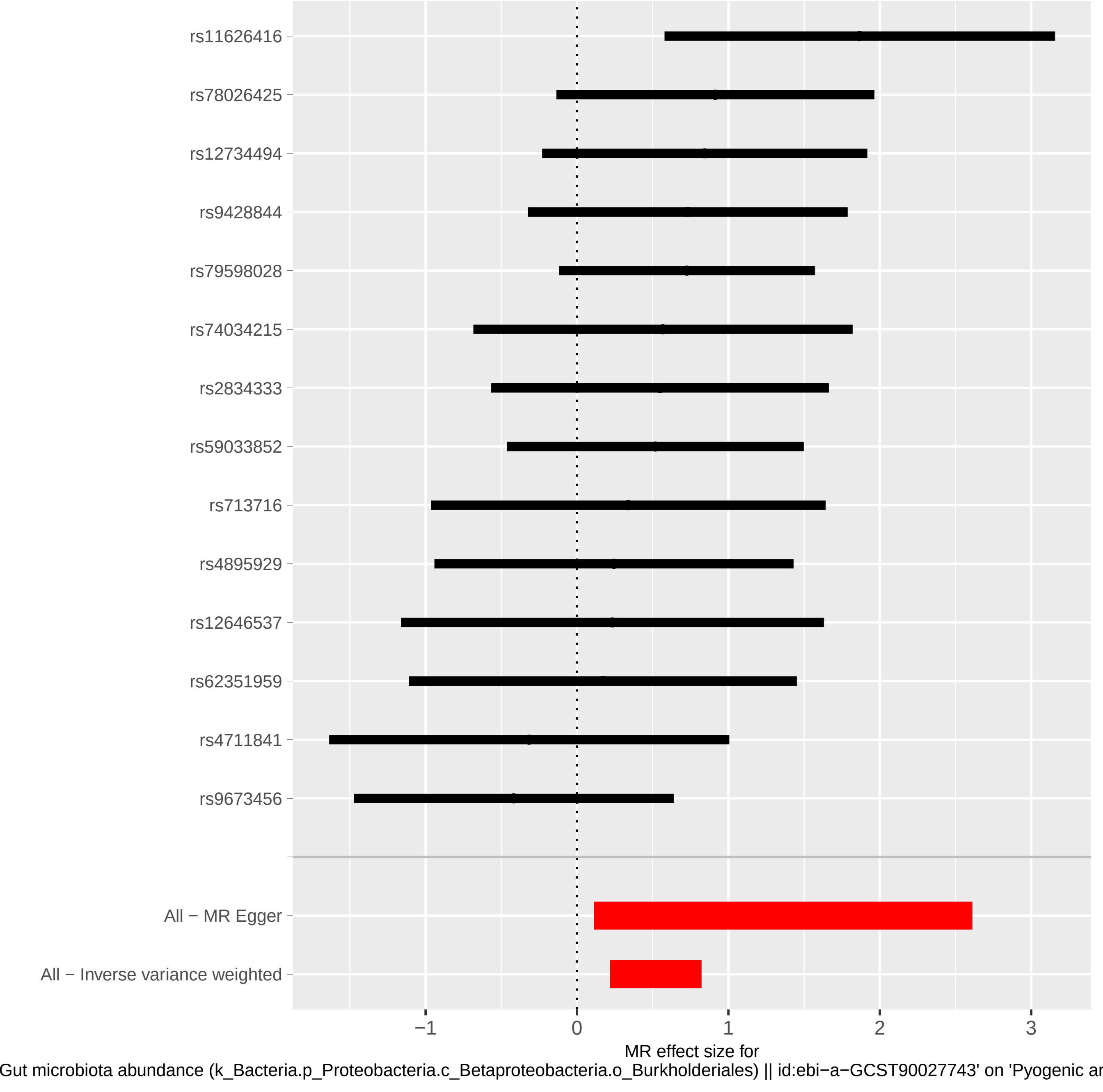

rs11626416
rs78026425
rs12734494
rs9428844
rs79598028
rs74034215
rs2834333
rs59033852
rs713716
rs4895929
rs12646537
rs62351959
rs4711841
rs9673456
All − MR Egger
All − Inverse variance weighted
−1
0
1
2
3
MR effect size for
Gut microbiota abundance (k_Bacteria.p_Proteobacteria.c_Betaproteobacteria.o_Burkholderiales) || id:ebi−a−GCST90027743' on 'Pyogenic ar
